# Generation of even and odd high harmonics in resonant metasurfaces using single and multiple ultra-intense laser pulses

**DOI:** 10.1038/s41467-021-24450-9

**Published:** 2021-07-07

**Authors:** Maxim R. Shcherbakov, Haizhong Zhang, Michael Tripepi, Giovanni Sartorello, Noah Talisa, Abdallah AlShafey, Zhiyuan Fan, Justin Twardowski, Leonid A. Krivitsky, Arseniy I. Kuznetsov, Enam Chowdhury, Gennady Shvets

**Affiliations:** 1 School of Applied and Engineering Physics, Cornell University, Ithaca, NY USA; 2 Institute of Materials Research and Engineering, A*STAR (Agency for Science, Technology and Research), Singapore, Singapore; 3 Department of Physics, The Ohio State University, Columbus, OH USA; 4 Department of Material Science and Engineering, The Ohio State University, Columbus, OH USA; 5 Department of Electrical and Computer Engineering, The Ohio State University, Columbus, OH USA

**Keywords:** Metamaterials, High-harmonic generation, Nonlinear optics, Photonic devices

## Abstract

High harmonic generation (HHG) opens a window on the fundamental science of strong-field light-mater interaction and serves as a key building block for attosecond optics and metrology. Resonantly enhanced HHG from hot spots in nanostructures is an attractive route to overcoming the well-known limitations of gases and bulk solids. Here, we demonstrate a nanoscale platform for highly efficient HHG driven by intense mid-infrared laser pulses: an ultra-thin resonant gallium phosphide (GaP) metasurface. The wide bandgap and the lack of inversion symmetry of the GaP crystal enable the generation of even and odd harmonics covering a wide range of photon energies between 1.3 and 3 eV with minimal reabsorption. The resonantly enhanced conversion efficiency facilitates single-shot measurements that avoid material damage and pave the way to study the controllable transition between perturbative and non-perturbative regimes of light-matter interactions at the nanoscale.

## Abstract (web summary)

Strong nonlinearities, like high harmonic generation in optical systems, can lead to interesting applications in photonics. Here the authors fabricate a thin resonant gallium phosphide metasurface capable of avoiding the laser-induced damage and demonstrate efficient even and odd high harmonic generation from it when driven by mid-infrared laser pulses.

## Introduction

Traditionally, high harmonic generation (HHG)^1–4^ has been observed in gases subjected to tunneling ionization by ultra-strong laser fields exceeding those that bind electrons to the nuclei^5,6^. High ionization thresholds, inversion symmetry, and infrastructure requirements imposed by gas chambers present challenges to the development of small-footprint low-power sources integrable in existing optoelectronic platforms for efficient and broadband HHG. Solid-state materials represent an attractive alternative for tabletop HHG sources^7,8^. However, conventional approaches to HHG utilizing bulk crystals fail to simultaneously achieve high conversion efficiencies and broad spectral bandwidth, owing to significant harmonics reabsorption and phase mismatch. More recently, designer nanostructures^9–16^ have attracted considerable attention, because they can potentially alleviate these problems due to locally enhanced optical “hotspot” fields through a variety of mechanisms: operation in the epsilon-near-zero regime^14^, high-quality-factor collective modes found in Si metasurfaces^11^, or plasmonic field enhancement^10^. However, several challenges to achieving highly efficient HHG in the strong-field regime assisted by spectrally selective metasurfaces can be identified. First, narrow- and moderate-bandgap semiconductors, with bandgap energies ${\Delta }_{{\rm{g}}}$ that are not much larger than the laser photon energy $\hslash \omega$, are damaged at moderate laser fluences due to multi-photon absorption followed by rapid free-carrier (FC) generation and recombination^17,18^. Moreover, the overabundance of FCs can drastically reduce the quality ($Q$) factor of a resonant metasurface^19^, thereby defeating its key purpose: the creation of resonantly driven optical hotspots. Second, harmonics absorption by opaque materials reduces the HHG-emitting volume and dramatically decreases the HHG efficiency^20^. Finally, only a subset of harmonics (odd) can be produced by centrosymmetric materials. Currently, non-centrosymmetric materials enabling even-order harmonics^13,21^ have not been utilized for nanostructure-based HHG: to date, high ($N\ge 4$) harmonics have only been reported from nanostructures biased by an external dc field^12^ or two-dimensional (2D) semiconductors^22^.

Therefore, it is desirable to develop a photonic platform and an optical system providing access to non-perturbative physics (defined by a strong perturbation by a laser pulse of the electron/hole motion in their respective conduction/valence bands)^22–25^. Here, a strong perturbation signifies electric fields large enough to violate the power series scaling law for the harmonics generation process. Such combination of a photonic platform and optical system must meet the following conditions: (a) the electronic bandgap of the constitutive material should be sufficiently large, so that multiple harmonic orders can be utilized; (b) the optical system should enable single-shot measurements that do not suffer from the inherent limitations of multi-pulse (MP) averaging, such as long-term damage^26–28^ and measurement biases (e.g., produced by a single high-intensity outlier in a train of laser pulses); and (c) the photonic structure should enable the production of nanoscale regions of a strongly driven material phase embedded inside a weakly perturbed phase, thus opening the possibility of studying nonlocal effects in condensed matter phase without confounding laser damage.

The transition to the non-perturbative nonlinear response occurs when the momentum gained from the laser electric field over a single period exceeds the size of the Brillouin zone of a solid material. This condition is expressed as $\beta \equiv {\omega }_{{\rm{B}}}{\left(2\omega \right)}^{-1} > 1$^8,23,29^, where ${\omega }_{{\rm{B}}}={eEa}{\hslash }^{-1}$ is the Bloch oscillation frequency^30–32^, $a$ a crystalline period, $\omega$ is the laser frequency, ℏ is the reduced Planck’s constant, and $E$ is the hotspot optical field. Concurrently, the injection of FCs into the conduction zone also takes place. The latter is governed by the dimensionless Keldysh parameter^33^$\gamma =\omega \sqrt{{m}^{\ast }{\Delta }_{{\rm{g}}}}{\left({eE}\right)}^{-1}$, where ${m}^{\ast }$ is the effective electron mass. Approximately equal to the ratio of the carrier injection time to laser period, the Keldysh parameter characterizes electron tunneling across the bandgap. Therefore, efficient non-perturbative (saturated) HHG requires that $\beta ,{\gamma }^{-1} > 1$.

Here we design and fabricate an ultrathin ($\approx 0.1\lambda$, where *λ* = 3.95 μm) resonant metasurface based on a transparent, high-index, wide-bandgap semiconductor: gallium phosphide (GaP)^34–36^. The combination of high refractive index ($n\approx 3$) and mid-infrared (MIR) transparency enables highly localized “hotspots” of the electromagnetic field inside GaP-based metasurfaces, akin to those made of silicon and gallium arsenide^37,38^. The large electronic bandgap (${\Delta }_{{\rm{g}}}^{({\rm{dir}})}=2.78$eV and ${\Delta }_{{\rm{g}}}^{({\rm{indir}})}=2.24{\rm{eV}}\gg \hslash \omega$) of GaP drastically reduces multi-photon absorption of MIR light (see Supplementary Information Section 1) and prevents visible HHG reabsorption up to the $N=7$ harmonic frequency ${\omega }_{\mathrm{\it{N}}}\equiv N\omega$. Finally, the non-centrosymmetric zincblende crystal structure of GaP enables generation of even-order harmonics from the bulk^13,25^. The key challenge addressed by our work is finding the appropriate photonic platform for entering this new regime without producing large numbers of FCs that can blue-shift^39^ and dampen^19^ the metasurface resonance. As illustrated by Supplementary Fig. 1 (see Supplementary Information Section 1 for the calculation of strong-field-induced FC generation), our choices of the metasurface material and laser wavelength $\lambda =2\pi c{\omega }^{-1}$ are strongly constrained if we are to access the non-perturbative regime of HHG in nanostructures. This selection of the laser wavelength and the underlying metasurface material enabled us to produce record-breaking unsaturated conversion efficiencies into high harmonics even in the perturbative regime of moderate laser intensity ${I}_{{{\max }}}^{({\rm{MP}})}\approx 80\,{\rm{GW}}\,{{\rm{cm}}}^{-2}$ in the MP illumination regime. By employing single-pulse (SP) measurements, we avoid laser-induced damage and reach the non-perturbative regime of HHG for incident laser intensities as high as ${I}_{{{\max }}}^{({\rm{SP}})}\approx 480\,{\rm{GW}}\,{{\rm{cm}}}^{-2}$. We observe a resonance-dependent saturation of the HHG at high estimated values of normalized Bloch oscillation frequencies ($\beta \approx 2$). The combination of the optical pulse format, metasurface design, and its constitutive material establishes a photonic platform for efficient non-perturbative light–matter interactions at the nanoscale and produces high-yield high harmonic radiation for applications in materials science and light sources.

## Results

### Sample design

**Fig. 1:**
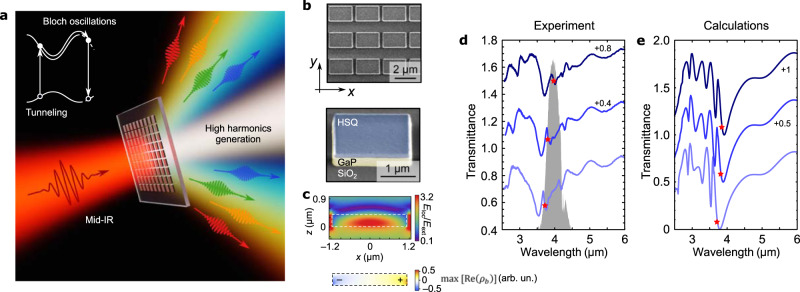
GaP metasurfaces for strong-field light–matter interactions in the mid-infrared. **a** Illustration of the high harmonic generation process: resonant GaP metasurfaces show efficient even and odd high harmonic generation (up to order H9) due to the wide direct electronic bandgap, high refractive index, non-centrosymmetric lattice, and intense-field-driven tunneling and Bloch oscillations. **b** Fabricated GaP metasurfaces: scanning electron microscope images, revealing the substrate (SiO_2_), the antenna material (GaP), and the lithography mask (hydrogen silsesquioxane, HSQ). **c** Calculated local field map ${E}_{{\rm{loc}}}{E}_{{\rm{ext}}}^{-1}$ of the metasurface mode excited by a mid-infrared (mid-IR) pulse with $\lambda ={\lambda }_{{\rm{res}}}^{(0)}$ and a corresponding induced bound charge map ${\rho }_{b}$ within the GaP antenna revealing an electric dipole mode. **d** Collimated (normal incidence) transmission spectra of three samples with varying dielectric resonant antenna sizes: largest (upper curve) to the smallest (lower curve) size. The second and third data sets are offset for clarity by +0.4 and +0.8, respectively. **e** COMSOL simulations of **d**. The second and third data sets are offset for clarity by +0.5 and +1.0, respectively. Red stars indicate the estimated wavelengths of the maximum local field enhancement.

The metasurfaces for enhanced HHG (Fig. 1a) were fabricated from 400 nm-thick GaP films using thin-film bonding, electron-beam lithography, and reactive ion etching (see Supplementary Information Section 2 for details). Figure 1b shows a scanning electron image of a typical metasurface sample. The metasurfaces consist of densely packed domino-shaped dielectric resonant antennas (DRAs) supporting externally excited resonant electric dipole (ED) electromagnetic modes at the nominal resonant wavelength ${\lambda }_{{\rm{res}}}^{(0)}=3.95\,\upmu {\rm{m}}$. These modes were experimentally identified for several metasurfaces with varying dimensions (and, correspondingly, varying resonant wavelengths ${\lambda }_{{\rm{res}}}={\lambda }_{{\rm{res}}}^{(0)}+\delta {\lambda }_{{\rm{res}}}$) using Fourier-transform infrared collimated beam spectroscopy^40^. At resonances—manifested as the transmission dips in the experimental (Fig. 1d) and numerical (Fig. 1e) spectra due to the excitation of the ED modes of the DRAs—metasurfaces funnel the MIR radiation into the “hotspots” (see Fig. 1c for a numerical simulation). The metasurface was nominally designed to provide moderate ${\left|\frac{{E}_{{\rm{loc}}}}{{E}_{{\rm{ext}}}}\right|}^{2}\approx 10$ intensity enhancement of the MIR radiation $\lambda ={\lambda }_{{\rm{res}}}^{(0)}$, with a dipole-type distribution of bound charge density ${\rho }_{{\rm{b}}}({\bf{r}},t)\propto (\varepsilon -1)\nabla {\bf{E}}({\bf{r}},t)$ shown in Fig. 1c at the peak of ${\rm{Re}}\left({\rho }_{{\rm{b}}}\right)$. The most efficient excitation of an ED mode occurs when its spectral bandwidth matches the MIR pump shown in Fig. 1d in gray.

### HHG measurements

**Fig. 2:**
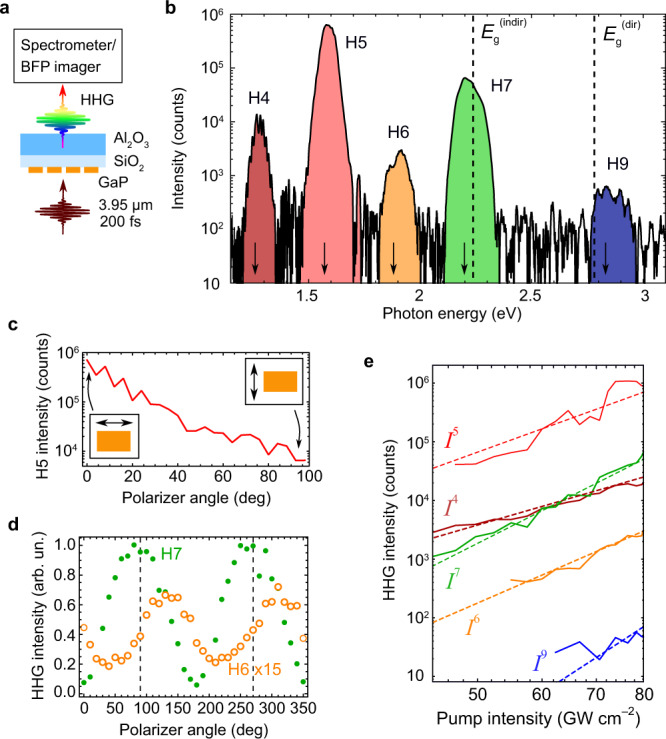
High harmonic generation in the perturbative multi-pulse (MP) regime. **a** Simplified schematic of the high harmonic generation (HHG) detection setup, with the detection arm represented by either a spectrometer or a back focal plane (BFP) imager. **b** MP-HHG spectra of the resonant sample at ${I}_{{\rm{MIR}}}=80\,{\rm{GW}}\,{{\rm{cm}}}^{-2}$. The *N* = 8 harmonic is not observed due to the onset of indirect interband transitions in GaP. The arrows indicate the predicted HHG wavelengths. **c** Polarization dependence of H5 shows two orders of magnitude contrast between the resonant (horizontal) and non-resonant (vertical) MIR polarizations with ${I}_{{\rm{MIR}}}=100\,{\rm{GW}}\,{{\rm{cm}}}^{-2}$. **d** Linear polarization of the odd-order (H7: green dots) and elliptic polarization of the even-order (H6: orange circles) harmonics. Dashed lines: MIR laser pulse polarization (${I}_{{\rm{MIR}}}=80\,{\rm{GW}}\,{{\rm{cm}}}^{-2}$). **e** Solid lines: HHG intensity as a function of the pump intensity for the *N* = 4 (dark red), *N* = 5 (red), *N* = 6 (orange), *N* = 7 (green), and *N* = 9 (blue) orders. Dashed lines: corresponding guide-to-the-eye power laws, ${I}^{(N)} \sim {I}_{{\rm{MIR}}}^{N}$.

Figure 2a shows a simplified sketch of the experimental setup for the detection and spectroscopy of HHG. Visible high harmonics emit from the metasurfaces driven by a femtosecond (${\tau }_{{\rm{MIR}}}\approx 200\,{\rm{fs}}$) pulse train centered at a wavelength $\lambda =3.95$ μm from an MIR optical parametric oscillator. The harmonics were detected via back focal plane (BFP) imaging or with a visible spectrometer (see Supplementary Information Sections 3–5 for details). A typical HHG spectrum, with the luminescence background subtracted, is shown in Fig. 2b. Even- and odd-order harmonics are observed in the near-infrared and visible ranges: from $\hslash {\omega }_{4}\approx 1.2{\rm{eV}}$ to $\hslash {\omega }_{9}\approx 3.0{\rm{eV}}$ (where ${\omega }_{\mathrm{N}}=2\pi {Nc}{\lambda }^{-1}$ is the *N*th harmonic frequency). No detectible harmonic signal was observed from either unstructured GaP film of the same thickness or the SiO_2_/Al_2_O_3_ substrate. The power of the seventh harmonic (H7) emitted from the sample was calibrated using an external laser source of a known power and a similar wavelength (see Supplementary Information Section 7 for the calibration procedure details). The absolute conversion efficiency reaches a value of ${\eta }_{7}\sim 2\times {10}^{-9}$ for H7 at $I=80\,{\rm{GW}}{{\rm{cm}}}^{-2}$, i.e., two orders of magnitude larger than the previous HHG demonstration in a metasurface^11^ and more than one order of magnitude larger than that in an epsilon-near-zero material^14^.

Crucially, even-order (H4 and H6) harmonics were detected alongside the odd-order harmonics (H5, H7, and H9) because of the non-centrosymmetric (zincblende) crystal structure of GaP. It is noteworthy that H8 was not detected in our experiment because of the combination of the indirect transitions at $\hslash {\omega }_{8}=2.28\,{\rm{eV}}$ (making GaP partially opaque at H8) and the inherently lower conversion efficiency of the even-order harmonics. The even harmonics’ relatively low efficiency can be attributed to the unfavorable orientation of the GaP principal crystalline axes inside the DRAs. The efficiency can be improved by about two orders of magnitude by a judicious choice of the crystal axis orientation (see Supplementary Information Section 8 and Supplementary Fig. 6).

To validate the importance of the dipole-active metasurface resonances, we have investigated the dependence of the H7 conversion efficiency on the polarization of the MIR pulse. The non-resonant pump polarization along the short side of the metasurface DRAs results in the efficiency reduction by two orders of magnitude compared with the resonant one, as shown in Fig. 2c. Therefore, the optical field enhancement inside the hotspot produced by the resonant laser polarization along the dipole moment of the ED mode is essential for the high efficiency of HHG observed in our experiments.

Next, we have analyzed the polarization states of the odd- and even-order harmonics. Specific examples for H7 and H6 harmonics are plotted in Fig. 2d for the (1, 0) diffraction order, as measured by BFP imaging. We observe that the odd harmonics (green dots) are co-polarized with the MIR pump (dashed lines). In contrast, the even harmonics (orange circles) are elliptically polarized, owing to the highly asymmetric structures of the even-order nonlinear susceptibility tensors ${\chi }_{{ij}\ldots k}^{\left(N\right)}$^41^, where the *N*th-order nonlinear polarization density of the medium is given by ${P}_{i}^{(N)}={\chi }_{{ij}\ldots k}^{\left(N\right)}{E}_{j}\ldots {E}_{k}$ (see Supplementary Information Section 8 and Supplementary Code 1 for details). For odd values of $N$, the diagonal matrix elements of ${\chi }_{{ij}\ldots k}^{\left(N\right)}$ dominate and the *N*th harmonic polarization is collinear with that of the MIR pump. In contrast, for even $N$, the elements of the ${\chi }_{{ij}\ldots k}^{\left(N\right)}$ tensor are predominantly off-diagonal, thereby enabling polarization changes of the even-order harmonics.

To investigate whether the HHG in the MP (moderate peak power) regime obeys the perturbative scaling laws, we have plotted in Fig. 2e the dependences of the harmonic intensity ${I}^{(N)}$ on the MIR intensity ${I}_{{\rm{MIR}}}$. The unsaturated dependences ${I}^{(N)} \sim {I}_{{\rm{MIR}}}^{N}$ are plotted as guides for the eye. In striking difference with the previous findings of HHG in nanostructures^11,14,42^, the GaP metasurface response does not exhibit any appreciable saturation. We conclude that the perturbative regime of harmonics generation persists up to the maximum pump intensity (${I}_{{\rm{MIR}}}\approx {I}_{{{\max }}}^{\left({\rm{MP}}\right)}=80\,{\rm{GW}}\,{{\rm{cm}}}^{-2}$) used in these experiments, which is equivalent to the hotspot intensity ${I}_{{\rm{hs}}}\approx 0.7\,{\rm{TW}}\,{{\rm{cm}}}^{-2}$ inside the metasurface. This agrees with our estimates of $\beta \,<\, 1$ for this range of intensities (see Supplementary Table 1).

### SP measurements

**Fig. 3:**
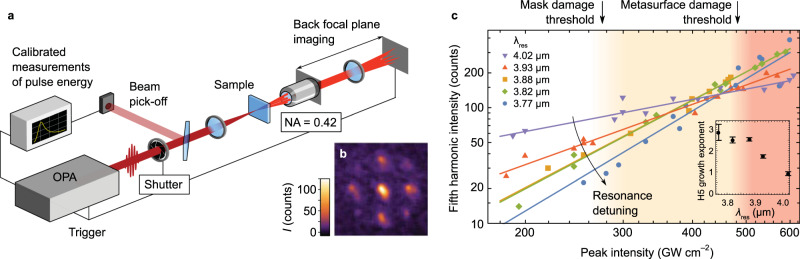
Single-pulse (SP) fifth harmonic generation reveals the non-perturbative regime and high damage thresholds of resonant metasurfaces. **a** A setup for SP-HHG back focal plane (BFP) imaging. Single pulses from an optical parametric amplifier (OPA) pass through a mechanical shutter, split into the main beam (sample irradiation) and the pick-off beam (individual pulse power calibration). The diffracted harmonics are collected by an objective lens with a numerical aperture of NA = 0.42 and detected in the BFP configuration by triggered camera exposure. **b** A typical BFP image of the H5 from the resonant sample at non-destructive intensities. **c** Zeroth diffraction order intensity of the H5 as a function of MIR pump intensity for five different metasurfaces with resonances at ${\lambda }_{{\rm{res}}}$, from the farthest from (blue circles) to the closest to (purple triangles) the driver wavelength. Solid lines: best fits to the power law ${I}^{(5)}=a{I}^{b}$. Deviation from the expected ${I}^{(5)} \sim {I}^{5}$ indicates the saturation of nonlinear response. Inset: power exponent $b$ vs. resonance wavelength ${{\rm{\lambda }}}_{{\rm{res}}}$. The mask damage threshold and the metasurface damage threshold are shown for the most resonant metasurface ${\lambda }_{{\rm{res}}}={\lambda }_{{\rm{res}}}^{(0)}=\lambda$.

As metasurfaces subjected to MP trains were visibly damaged for incident laser intensities of the order ${I}_{{\rm{MIR}}}\approx 200\,{\rm{GW}}\,{{\rm{cm}}}^{-2}$, the only non-destructive pathway to accessing the non-perturbative laser–matter interaction regime is to resort to SP experiments. Moreover, unlike MP averaging that may not provide the complete picture of nonlinear processes, the SP exposures yield accurate relationships between the pulse energy, HHG signal, and the excitation site within the sample, while avoiding the accumulation of MP damage^26–28^. To access the high-intensity regime ($0.2\mbox{-}0.6\,{\rm{TW}}\,{{\rm{cm}}}^{-2}$), we replaced the focusing optics and synchronized the elements of the setup. As schematically shown in Fig. 3a, the optical parametric amplifier (OPA) triggers a mechanical shutter, directing a single laser pulse to the sample and into the pick-off detection arm. The sample resides on a three-dimensional translation stage and is monitored by a visible-light imaging system (not shown). Each area of the sample is exposed to a single laser pulse by moving it out of the laser path by 50 μm after each shot. For each shot, the trigger starts the fast camera acquisition that records BFP images of the HHG pattern; a typical single-shot BFP image is shown in Fig. 3b.

As an example, the zeroth diffraction order is plotted as a function of the field intensity in Fig. 3c for five different metasurfaces, from the one with the smallest detuning between the pump and the resonance (purple triangles: ${{\rm{\lambda }}}_{{\rm{res}}}={{\rm{\lambda }}}_{{\rm{res}}}^{(0)}$) to the largest detuning (blue circles). The solid lines show the best fits to the power law ${I}^{(5)} \sim a{I}^{b}$, where the exponent $b$ is expected to be equal to 5 for the perturbative H5 process. However, in contrast with moderate-intensity data in Fig. 2e, the drastic reduction of the H5 exponent ($b \,<\, 5$) signifies the onset of the non-perturbative regime. The inset of Fig. 3c shows $b({\lambda }_{{\rm{res}}})$ as a function of the detuning between the incident pulse and the resonant wavelength ${\lambda }_{{\rm{res}}}$. The exponent $b$ varies monotonically between $b=2.8$ for the least resonant metasurfaces to $b=0.9$ for the most resonant metasurface.

The scanning electron micrographs of the degraded metasurfaces reveal two types of damage caused by the single pulses: the mask damage for ${I}_{{\rm{MIR}}} \,> \, {I}_{{{\max }}}^{({\rm{HSQ}})}\approx 280\,{{\rm{GW}}\,{\rm{cm}}}^{-2}$ (detachments of the hydrogen silsesquioxane (HSQ) cap from the GaP resonators) and the structure damage for ${I}_{{\rm{MIR}}} > {I}_{{{\max }}}^{({\rm{GaP}})}\approx 480\,{{\rm{GW}}\,{\rm{cm}}}^{-2}$ (removal of the GaP resonators from the substrate). Surprisingly, even though well-defined damage thresholds are identified by observing the metasurface degradation, no abrupt changes in HHG are experimentally observable at those threshold intensities ${I}_{{{\max }}}^{({\rm{HSQ}})}$ and ${I}_{{{\max }}}^{({\rm{GaP}})}$ (see Fig. 3c). The primary mechanism for the damage is the photo-mechanical spallation of GaP layer beneath the high-bandgap HSQ layer, induced by the highly excited FCs in GaP (see Supplementary Information Section 6 for further discussion). The lack of any abrupt changes in the HHG dependences is attributed to the beam’s finite size: the HHG output is maintained at the beam’s periphery even when the centrally positioned portion of the sample is damaged by a laser pulse. The estimated conversion efficiency of H5 in the SP regime at $I=200\,{\rm{GW}}\,{{\rm{cm}}}^{-2}$ for sample #5 (resonant case) is ${\eta }_{5\omega }=1.4\times {10}^{-6}$, which is almost two orders of magnitude larger than that in the MP case. A comparison between various solid-state HHG sources, provided in Supplementary Table 2, shows that the GaP metasurface provides the largest specific (per unit length) conversion efficiency among all the materials provided.

## Discussion

One of the primary mechanisms contributing to the HHG in the non-perturbative regime is the generation of the nonlinear currents by the Bloch oscillations^31^ of the FCs. The local (hotspot) field strength that does not destroy the most resonant GaP metasurface (corresponding to ${I}_{{\rm{MIR}}}\approx {I}_{{{\max }}}^{({\rm{GaP}})}$) can be estimated to be ${E}_{{{\max }}}^{({\rm{hs}})}\approx 0.24\,{\mathrm{V}}{\mathring{\rm{A}} }^{-1}$ (assuming a factor ${\rm{\times }}10$ intensity enhancement at the hotspot), bringing the value of the Bloch frequency up to ${\omega }_{{\rm{B}}}\approx 2{\rm{\times }}{10}^{15}$ s^–1^. The corresponding ratio of the Bloch frequency to the driving MIR laser frequency is $\beta ={\omega }_{{\rm{B}}}{\left(2\omega \right)}^{-1}\approx 2.1$, thus suggesting a transition to a non-perturbative response of the underlying GaP crystal (see Supplementary Table 1). The anisotropic response of the electron subsystem suggests the importance of crystal lattice orientation, whereby one can tailor the contributions from different harmonics by engineering the crystal axes within the nanostructure. These are promising topics for future studies.

In conclusion, we have demonstrated efficient visible high harmonics generation using MIR resonances in ultrathin GaP metasurfaces. Our approach provides record-high conversion efficiency at the nanoscale, enabled by the combination of strong hotspot enhancement of the optical field, high resilience of the underlying material to strong fields, and the low level of HHG reabsorption. The SP illumination format enabled us to utilize much higher laser intensities than in the multiple-pulse format, thereby accessing the non-perturbative regime of HHG without confounding structural damage. The robustness of the metasurface to laser damage under ultra-intense illumination facilitates strong-field regimes with engineered light fields and enables non-perturbative light–matter interactions at the micro- and nanoscales.

## Methods

### Sample fabrication

Crystalline GaP layer (∼400 nm) is first grown on a GaAs substrate with an AlGaInP buffer layer by metal-organic chemical vapor deposition. Then, this structure is directly bonded to a sapphire substrate (150 μm) after depositing ∼2 μm SiO_2_ layers on top of both surfaces. The AlGaInP/GaAs substrate is then removed by wet etching. The fabrication of the GaP nanostructures starts with a standard wafer cleaning procedure (using acetone, isopropyl alcohol, and deionized water in that sequence under sonication), followed by O_2_ and hexamethyl disilizane priming, to increase the adhesion between GaP and subsequent spin-coated electron-beam lithography (EBL) resist of HSQ. After spin-coating of HSQ layer with a thickness of ∼200 nm, EBL and development in 25% tetra-methyl ammonium hydroxide were carried out to define the patterns in the HSQ resist. Finally, inductively coupled plasma reactive ion etching with N_2_ and Cl_2_ gases was used to transfer the HSQ patterns to the GaP layer and generate the GaP nanostructures. The orientation of the GaP crystal lattice with respect to the metasurface is visualized by orienting the [001] direction perpendicular to the plane of metasurface and then tilting the normal with respect to the plane metasurface by 15° toward the [111] direction of the GaP crystal lattice.

### HHG measurements

In Supplementary Fig. 2a, detailed schematic of the optical setup used for HHG is shown. The Extreme MIR (EMIR) OPA is a home-built KNbO_3_/KTA 3-crystal/3-pass OPA. EMIR is pumped by The Ohio State University’s GRAY laser, a home-built 80 fs Ti:Sapphire chirped pulse amplification system with a central wavelength of 780 nm and 4 mJ per pulse. For the experiments, the MIR (idler) beam was fixed at $\lambda =3.95\,{{\upmu }}{\rm{m}}$ and collimated to a size of about 2.5 mm. Output modes were characterized for several different wavelengths using a WinCamD-FIR2-16-HR 2–16 μm Beam Profiler System. The MIR pulse duration was measured using an AgGaS_2_-crystal-based MIR autocorrelator for 3 and 3.6 μm to be $\tau =200$ fs. The MIR spectra were obtained using an A.P.E. Wavescan USB MIR spectrometer. The MIR pulse energy was controlled with a half-wave plate–wire-grid polarizer pair in the range of about 1–6 μJ. Upon transmission through the sample, the upconverted signal was collected with a large-working distance (20 mm) Mitutoyo objective (numerical aperture = 0.42), which allows collection of the transmitted harmonics and several diffraction orders. The BFP of the objective was projected onto the sensor of a thermoelectrically cooled back-illuminated charge-coupled device (CCD) camera (Princeton Instruments PIXIS 1024 BUV). To spectrally filter the individual optical harmonics for BFP imaging, a set of long-, short-, and band-pass filters (Thorlabs FEL and FESH series, FGB37) was used. Supplementary Fig. 3 shows the BFP images of harmonics H4, H5, H6, H7, and H9 after spectral filtration, exposing the diffraction patterns, as well as the incoherent luminescence that fills the whole aperture of the objective for some wavelength ranges.

For HHG spectra acquisition, the BFP of the objective lens was projected onto the entrance slit of a monochromator (Chromex 250SM scanning monochromator) coupled to the same CCD camera. In a typical raw spectroscopic image (see Supplementary Fig. 4), the left image shows the camera output in the spectral range capturing H6 and H7, where both can be discerned on top of the luminescence background. To subtract the incoherent background, for each wavelength, we fit the *y*-section of the image to a Gaussian near the zeroth order diffraction (middle 30 pixels). For Fig. 2b, the amplitude of the Gaussian is plotted to separate the HHG signal from the luminescence background as a function of the wavelength.

To reach the non-perturbative intensities with our experiments, we added a functionality for the setup to be able to irradiate the sample with individual laser pulses at substantially higher intensities than those used in MP measurements. The focusing lens was changed to one with *f* = 50 mm and the beam size was measured to be an ellipse with axes $\Delta {x}_{{\rm{FWHM}}}=53\,{\upmu }{\rm{m}}$ and $\Delta {y}_{{\rm{FWHM}}}=43\,{\upmu }{\rm{m}}$ by putting a 2D micro-bolometer array sensor (DataRay WinCamD-IR-BB, pixel pitch 17 μm) in the focal point of an attenuated beam. For the SP acquisition, the repetition rate of the laser was lowered to 10 Hz and the measurements were done in the BFP setting with triggered exposure. The software-controlled trigger from the laser was sent to the mechanical shutter (1/30 s opening time) and an oscilloscope that received the signal from an amplified PbS photodiode that detected the energy of a pick-off pulse. The diode was calibrated using a pyroelectric power meter (Gentek-EO QE-B), averaging over 5000 pulses for each power setting in the range from 0.5 to 5 μJ. As the fluences used in these experiments lie close to the SP damage threshold of the sample, we chose a fresh spot of the metasurface for each shot, moving at least 50 μm away between the shots.

## Supplementary information

Supplementary Information

Description of Additional Supplementary Files

Supplementary Code 1

## Data Availability

The data that support the findings of this study are available from the corresponding author upon reasonable request.

## Code availability

The code that was generated and used during this study is available from the corresponding author upon reasonable request.
