# Supplementary material for: Generation of even and odd high harmonics in resonant metasurfaces using single and multiple ultra-intense laser pulses
Source: Nat Commun. 2021 Jul 7;12:4185. doi: 10.1038/s41467-021-24450-9 (PMC8263774; doi:10.1038/s41467-021-24450-9)
Supplement: Supplementary file 1 — Supplementary Information [file 41467_2021_24450_MOESM1_ESM.pdf]

Generation of even and odd high harmonics in resonant metasurfaces using single and multiple ultra-intense laser pulses. Supplementary information

Authors: Maxim R. Shcherbakov,[1,*] Haizhong Zhang,[2] Michael Tripepi,[3] Giovanni Sartorello,[1] Noah Talisa,[3] Abdallah AlShafey,[3] Zhiyuan Fan,[1] Justin Twardowski,[3] Leonid A. Krivitsky,[2] Arseniy I. Kuznetsov,[2] Enam Chowdhury,[3,4,5] Gennady Shvets[1,*]

Affiliations:

[1]School of Applied and Engineering Physics, Cornell University, Ithaca, NY 14853, USA.

[2]Institute of Materials Research and Engineering, A*STAR (Agency for Science, Technology and Research), 138634, Singapore.

[3]Department of Physics, The Ohio State University, Columbus, OH 43210, USA.

[4]Department of Material Science and Engineering, The Ohio State University, Columbus, OH 43210, USA.

[5]Department of Electrical and Computer Engineering, The Ohio State University, Columbus, OH 43210, USA.

*Correspondence to: mrs356@cornell.edu, gshvets@cornell.edu

1. Experimental parameter and ionization rates estimates

Following the estimates of the field intensity and the temporal local field enhancement factor $L \approx 3$ (defined as $E = LE_{\mathrm{ext}}$, where E is the peak value of the field within the GaP resonator, E_{ext} is the external field), we can provide estimates of the peak fields within the structure, as well as the general metrics of the light-matter interactions in the metasurface. In Supplementary Table 1, three values of field intensity are given: the maximum multi-shot intensity used in Fig. 2e, minimum single-shot intensity used in Fig. 3 of the main text, and the intensity corresponding to the middle point between the damage thresholds of the mask and the resonators. The columns show the following calculated quantities: vacuum intensity I_{vac}, local intensity $I = L^2 I_{\mathrm{vac}}$, electric field strength $E = \sqrt{I[\mathrm{TW\ cm^{-2}}]}\ 0.137\ \mathrm{V\ Å^{-1}}$; the Bloch oscillation frequency $\omega_{\mathrm{B}} = |eE|a\hbar^{-1}$, where $e = 1.6\ \times 10^{-19}$ C, $a\ =\ 5.44$ Å is the GaP lattice constant, $\hbar = 1.05 \times 10^{-34}$ J s is the reduced Planck's constant; β parameter, $\beta = \omega_{\mathrm{B}}(2\omega)^{-1}$, where $\omega = 4.77 \times 10^{14}\ \mathrm{s^{-1}}$ is the pump frequency; δ parameter, $\delta = E/E_{\mathrm{crit}}$, where the critical field $E_{\mathrm{crit}} = \Delta_{\mathrm{g}}(ea)^{-1}$ and $\Delta_{\mathrm{g}} = 2.78$ eV is the Γ-point gap; the Keldysh parameter $\gamma = \omega\sqrt{m^*\Delta_{\mathrm{g}}}(eE)^{-1}$, where $m^* = 0.09m$ is the Γ-valley electron mass, $m\ =\ 9.1 \times 10^{-31}$ kg.

I_{vac} (TW cm^{-2})	I (TW cm^{-2})	E (V Å^{-1})	ω_{B} (10^{14} s^{-1})	$\beta = \frac{\omega_B}{2\omega}$	$\delta = \frac{E}{E_{\mathrm{crit}}}$	γ
0.07	0.6	0.11	9	0.9	0.21	0.52
0.2	1.8	0.18	15	1.6	0.36	0.31
0.33	3	0.24	20	2.1	0.46	0.24

Supplementary Table 1 | Light-matter interaction metrics in gallium phosphide metasurfaces. I_{vac} is the MIR pump intensity in a vacuum near the focal spot ($\lambda = 3.95$ μm), I is the pump intensity at the metasurface's hot spot, E is the MIR pump field strength at the metasurface's hot spot, $\omega_{\mathrm{B}} = aeE\hbar^{-1}$ is the corresponding Bloch frequency, $E_{\mathrm{crit}} = e\Delta_{\mathrm{g}}a^{-1}, \gamma = \omega\sqrt{m^*\Delta_{\mathrm{g}}}(eE)^{-1}$ is the Keldysh parameter. Note that for high field strengths, the effective mass approximation may not provide reliable values of γ.

To estimate the ionization rates in a material with the ionization potential Δ_g by an ac field at frequency ω and strength E, we apply Perelomov–Popov–Terent'ev theory[1] which describes both the multiphoton and tunneling ionization processes. In this model, time-dependent Schrodinger equation is analytically solved in the quasi-static, single active electron approximation. We estimate the ionization rate using the following solution:

$$W \approx \frac{\Delta_g}{\hbar}\left(\frac{E\sqrt{1+\gamma^2}}{2E_0}\right)^{\frac{3}{2}} A(\omega,\gamma)e^{-\frac{2E_0}{3\,E}g(\gamma)},$$

where $A(\omega,\gamma) = \gamma^2/(1+\gamma^2)\sum_{n\le\nu}\exp[-\alpha(\gamma)(n-\nu)]\,w[\sqrt{\beta(n-\nu)}]$, $\alpha = 2(\operatorname{arcsinh}\gamma - \gamma/\sqrt{1+\gamma^2})$, $\beta = 2\gamma/\sqrt{1+\gamma^2}$, $w(x) = \frac{x}{2}\int_0^1 \exp(-x^2 t)\, t/\sqrt{1-t}dt$, $g = 3/2\gamma\left[(1+1/2\gamma^2)\operatorname{arcsinh}\gamma - \sqrt{1+\gamma^2}/2\gamma\right]$, and E_0 is the characteristic field of an electron in a state with binding energy E_g. Calculated rates W are multiplied by the pulse duration $t = 100$ fs to estimate the ionization probability by a single pulse $P = Wt$, which is plotted in Supplementary Fig. 1 as a function of the excitation field frequency for various values of $\beta = aeE(2\hbar\omega)^{-1}$, where $a = 5.44$ Å is the crystal lattice constant. The regime with $\beta > 1$, whereby Bloch oscillations can meaningfully contribute to the process of HHG, is denoted by the shaded pink area. We have also estimated the ionization probability that causes more than $\rho_{\text{crit}} = 10^{19}$ cm^{-3} electron-hole pairs at $P_{\text{crit}} = \rho_{\text{crit}}a^3/8 \approx 10^{-4}$, where 8 stands for the number of atoms in the unit cell, as marked by the dashed line in Supplementary Fig. 1. The plasma as dense as ρ_{crit} can cause significant free-carrier-induced absorption and deteriorate both the resonance of the metasurface and its HHG output. The circle indicates the estimated experimental conditions ($\beta \approx 2, \hbar\omega/\Delta_g \approx 0.12$), which signify an important physical regime, whereby an ultra-intense laser pulse can lead to non-perturbative response without actively damaging the material or

causing sub-optimal nonlinear response hindered by the generated free carriers. The family of dashed curves plotted for a narrower band gap of 1 eV show a considerably higher ionization, which may prevent efficient HHG in narrow-gap semiconductors. It is important to note that, even though the PPT model is not as accurate as the Keldysh model,[2] because it does not take into account any details of the electronic band structure, we have verified that the two model are in approximate agreement for the case of GaP, as the Keldysh model provides us with similar ionization rates for the parameters we used in our experiment.

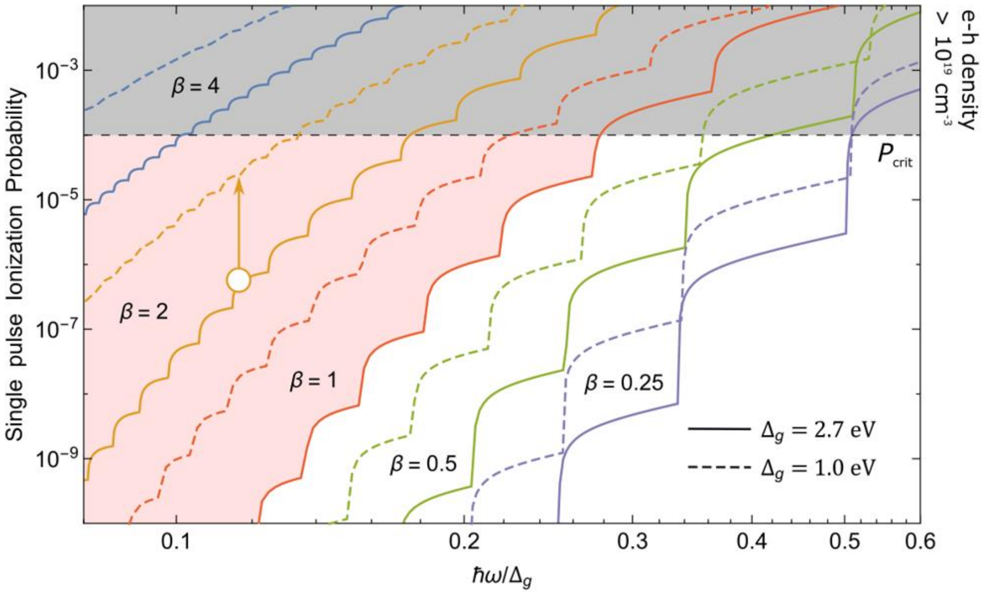

Supplementary Fig. 1 | Single-pulse ionization probability estimates. A system with ionization potential $\Delta_g = 2.7$ eV is excited by $t = 100$ fs pulse with a photon energy $\hbar\omega$ and peak electric field E for various values of $\beta = 4, 2, 1, 0.5, 0.25$ (blue, orange, red, green and purple solid curves, respectively). The circle denotes the estimated experimental conditions, the shaded pink area denotes the region of strong-field ($\beta > 1$) excitation and the gray area denotes the dense photon-induced e-h plasma regime ($\rho > 10^{19}$ cm^{-3}). The dashed lines show the same family of curves for $\Delta_g = 1.0$ eV.

2. Sample fabrication

Crystalline GaP layer (∼400 nm) is first grown on a GaAs substrate with an AlGaInP buffer layer by metal-organic chemical vapor deposition (MOCVD). Then this structure is directly bonded to a sapphire substrate (150 μm) after depositing ∼2 μm SiO_2 layers on top of both surfaces. The AlGaInP/GaAs substrate is then removed by wet etching. The fabrication of the GaP nanostructures starts with a standard wafer cleaning procedure (using acetone, isopropyl alcohol and deionized water in that sequence under sonication), followed by O_2 and hexamethyl disilizane (HMDS) priming in order to increase the adhesion between GaP and subsequent spin-coated electron-beam lithography (EBL) resist of hydrogen silsesquioxane (HSQ). After spin-coating of HSQ layer with a thickness of ∼200 nm, EBL and development in 25% tetra-methyl ammonium hydroxide (TMAH) were carried out to define the patterns in the HSQ resist. Finally, inductively coupled plasma reactive ion etching (ICP-RIE) with N_2 and Cl_2 gases was used to transfer the HSQ patterns to the GaP layer and generate the GaP nanostructures; see Fig. 1b for scanning electron micrographs (SEM) of the sample's fragment. The orientation of the GaP crystal lattice with respect to the metasurface is visualized by orienting the [001] direction perpendicular to the plane of metasurface, and then tilting the normal to the metasurface' plane by 15° toward the [111] direction of the GaP crystal lattice.

3. High harmonic measurement setup

In Supplementary Fig. 2, a detailed schematic of the optical setup used for high harmonic generation is shown. The Extreme Mid-IR (EMIR) optical parametric amplifier (OPA) is a homebuilt $KNbO_3$/KTA 3-crystal/3-pass OPA. EMIR is pumped by The Ohio State University's GRAY laser, a homebuilt 80-fs Ti:Sapphire chirped pulse amplification system with a central

wavelength of 780 nm and 4 mJ per pulse. The repetition rate of EMIR can be varied nearly continuously between 1 and 500 Hz using an external Pockels-cell-based pulse picker. EMIR can generate 200-fs mid-IR pulses with up to 25 μJ per pulse. The output wavelength of EMIR can be varied continuously from $\lambda = 2.7$ μm to 4.5 μm. For the experiments, the MIR (idler) beam was fixed at $\lambda = 3.95$ μm and collimated to a size of about 2.5 mm. Output modes were characterized for several different wavelengths using a WinCamD-FIR2-16-HR 2 to 16μm Beam Profiler System. The MIR pulse duration was measured using an AGS-crystal-based MIR autocorrelator for 3 and 3.6 μm to be $\tau = 200$ fs. The MIR spectra were obtained using an A.P.E. Wavescan USB MIR spectrometer. The MIR pulse energy was controlled with a half-wave plate—wire-grid polarizer pair in the range of about 1 to 6 μJ.

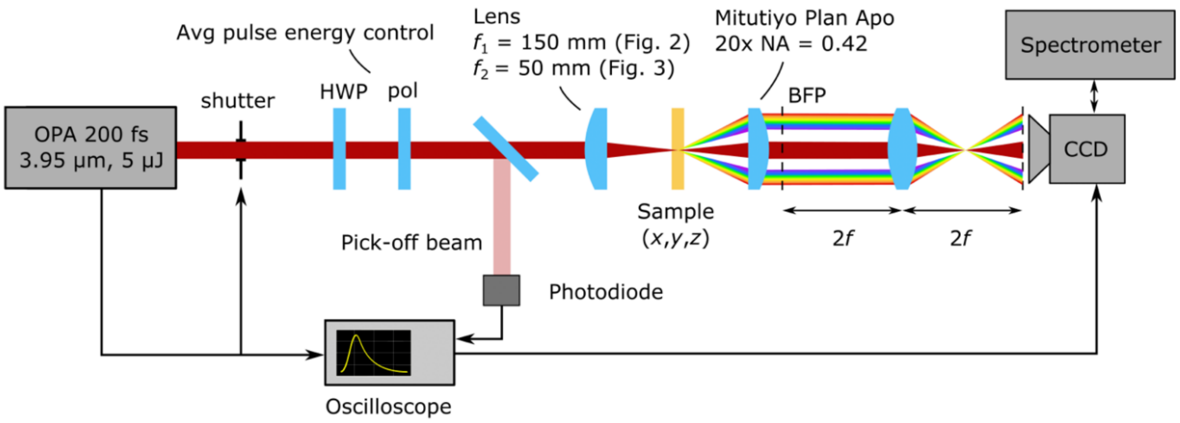

Supplementary Fig. 2 | High harmonic generation setup. OPA — optical parametric amplifier, HWP — half-wave plate, pol — wire-grid polarizer, BFP — back focal plane of the collecting objective.

4. Beam parameters

The beam spot at the sample plane was characterized using the same beam profiler system. For multi-pulse (perturbative) measurements, a CaF_2 lens with a focal distance of $f_{\mathrm{MP}}^{(1)} = 150$ mm was used; the focal spot size was measured to be $\Delta x_{\mathrm{FWHM}}^{(1)} = 175$ µm by $\Delta y_{\mathrm{FWHM}}^{(1)} = 153$ µm (intensity full width at half-maximum). For single-pulse (non-perturbative) measurements, a lens with a focal spot of $f_{\mathrm{SP}}^{(2)} = 50$ mm was used; the focal spot size was measured to be $\Delta x_{\mathrm{FWHM}}^{(2)} = 53$ µm by $\Delta y_{\mathrm{FWHM}}^{(2)} = 43$ µm (intensity full width at half-maximum). The peak intensity I_0 within the focal spot can be estimated approximating the beam profile with a 2D Gaussian:

$$W = I \int \exp\left(-\frac{4\ \mathrm{ln2}\ x^2}{\Delta x_{\mathrm{FWHM}}^2} - \frac{4\ \mathrm{ln2}\ y^2}{\Delta y_{\mathrm{FWHM}}^2}\right) dxdy,$$

Where W is the pulse power. The energy-to-peak-intensity conversion coefficient is therefore expressed as:

$$K = \frac{I}{E} = \frac{4\ \mathrm{ln2}}{\pi \Delta x_{\mathrm{FWHM}} \Delta y_{\mathrm{FWHM}} \tau},$$

with the estimated values of $K_{\mathrm{SP}}^{(1)} = 1.65 \times 10^{16}\ \mathrm{s}^{-1}\mathrm{cm}^{-2}$ for multi-pulse measurements and $K_{\mathrm{MP}}^{(2)} = 1.94 \times 10^{17}\ \mathrm{s}^{-1}\mathrm{cm}^{-2}$ for single pulse-measurements. This way, the full range of accessed intensities ranging from 40 GW cm^{-2} to 80 GW cm^{-2} in the multi-pulse measurements and from 200 GW cm^{-2} to 600 GW cm^{-2} in the single-pulse measurements.

5. HHG measurements

Upon transmission through the sample, the upconverted signal was collected with a large-working distance (20 mm) Mitutoyo objective (NA = 0.42) which allows collection the transmitted harmonics as well as several diffraction orders. The back focal plane of the objective

was projected onto the sensor of a thermoelectrically cooled back-illuminated CCD camera (Princeton Instruments PIXIS 1024 BUV). To spectrally filter the individual optical harmonics for back focal plane imaging, a set of long-, short- and band-pass filters (Thorlabs FEL and FESH series, FGB37) was used. Supplementary Fig. 3 shows the back focal plane images of harmonics H4, H5, H6, H7, H9 after spectral filtration, exposing the diffraction patterns, as well as the incoherent luminescence that fills the whole aperture of the objective for some wavelength ranges. Exposure times were 3 s, 100 ms, 10 s, 3 s, 10 s, respectively.

For HHG spectra acquisition, the back focal plane of the objective lens was projected onto the entrance slit of a monochromator (Chromex 250SM scanning monochromator) coupled to the same CCD camera. In a typical raw spectroscopic image, see Supplementary Fig. 4, the left image shows the camera output in the spectral range capturing H6 and H7, where both can be discerned on top of the luminescence background. In order to subtract the incoherent background, for each wavelength, we fit the y-section of the image to a Gaussian near the zeroth order diffraction (middle 30 pixels). For Fig. 2(b) of the main text, the amplitude of the Gaussian is plotted to separate the HHG signal from the luminescence background as a function of the wavelength. Figure 2(b) of the main text consists of measurements from six separate spectrometer windows for each of the harmonics (H4, H5, H6, H7, H8, H9). For each window, a specific exposure time was selected, based on the signal level of the harmonic. The chosen exposure times were 3 s, 100 ms, 10 s, 3 s, 10 s, 10 s; within each window, the measured intensity spectra were divided by the corresponding exposure times and plotted within the same axis. Note that at this point, Fig. 2(b) is not a calibrated spectrum, and none of the spectrally sensitive elements of the setup have been accounted for.

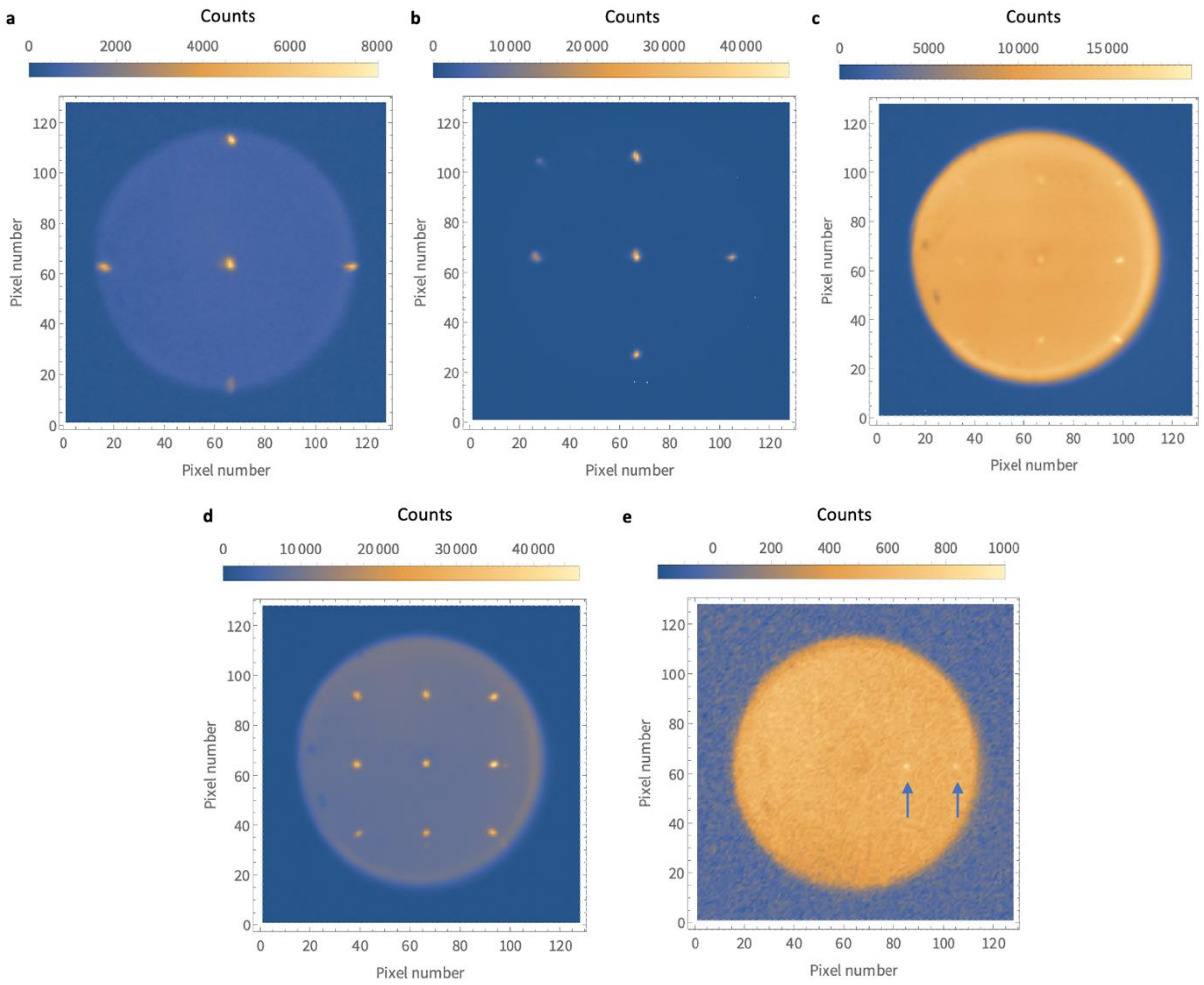

Supplementary Fig. 3 | Back focal plane images of harmonics within different spectral bands. a, $\lambda_{\text{pass}} > 900$ nm ($\lambda_{\text{H4}} \approx 990$ nm). **b,** 700 nm $< \lambda_{\text{pass}} <$ 850 nm ($\lambda_{\text{H5}} \approx 790$ nm). **c,** 600 nm $< \lambda_{\text{pass}} <$ 700 nm ($\lambda_{\text{H6}} \approx 660$ nm). **d,** 500 nm $< \lambda_{\text{pass}} <$ 700 nm ($\lambda_{\text{H7}} \approx 560$ nm), **e,** 350 nm $< \lambda_{\text{pass}} <$ 500 nm ($\lambda_{\text{H9}} \approx 430$ nm); arrows indicate the visible H9 diffraction orders. In some of the images, the luminescence background is present, filling the whole back focal plane of the objective.

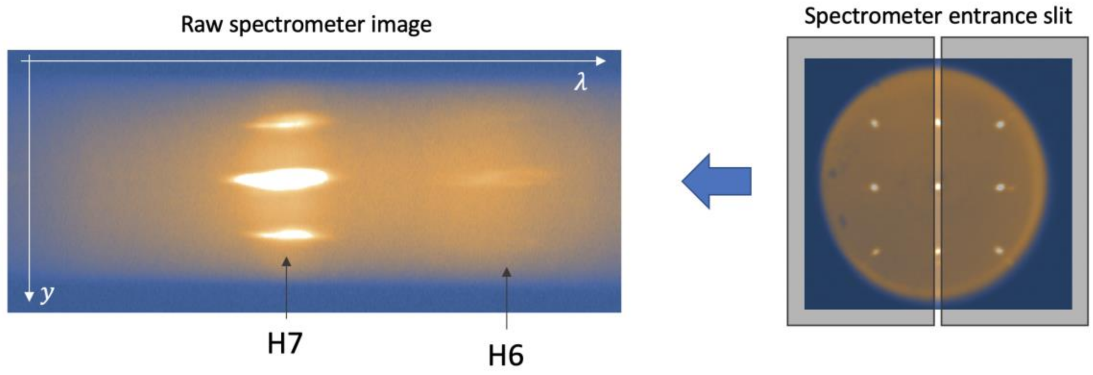

Supplementary Fig. 4 | HHG spectroscopy schematic. The back focal plane image of the sample was projected onto the entrance slit of the monochromator (right) resulting in the two-dimensional image at the camera after the monochromator (left). On the raw image, H7 and H6 are visible, both their (0; 0) and $(0; \pm 1)$ orders, as well as the broadband luminescence spectrum.

6. Single-shot HHG and damage threshold measurements

To reach the non-perturbative intensities with our experiments, we added a functionality for the setup to be able to irradiate the sample with individual laser pulses the emitted harmonics generation at substantially higher intensities than those used in multi-pulse measurements. The focusing lens was changed to one with $f = 50$ mm, and the beam size was measured to be an ellipse with axes $\Delta x_{\mathrm{FWHM}} = 53$ μm and $\Delta y_{\mathrm{FWHM}} = 43$ μm by putting a 2D micro-bolometer array sensor (DataRay WinCamD-IR-BB, pixel pitch 17 μm) in the focal point of an attenuated beam. For the single-pulse acquisition, the repetition rate of the laser was lowered to 10 Hz, and the measurements were done in the back focal plane setting with triggered exposure. The software-controlled trigger from the laser was sent to the mechanical shutter (1/30 s opening time) and an oscilloscope that received the signal from an amplified PbS photodiode that detected the energy of a pick-off pulse. The diode was calibrated using a pyroelectric power meter (Gentek-EO QE-B), averaging over 5000 pulses for each power setting in the range from

0.5 μJ to 5 μJ. Since the fluences used in these experiments lie close to the single-pulse damage threshold of the sample, we chose a fresh spot of the metasurface for each shot, moving at least 50 μm away between the shots. As an example, Supplementary Fig. 5 shows the resonant metasurface that has been exposed by single pulses in the energy ranges from 0.5 μJ to 3.8 μJ. We have divided the outcomes of single-pulse irradiation into three scenarios: state '0' with no apparent damage done to the sample, state '1' with the HSQ mask getting detached (as supported by the SEM images) and state '2' with the GaP resonators partially removed from the substrate. Supplementary Fig. 5 shows the dependence of the outcome on the measured single-pulse energy. The importance of the single-pulse measurements is pinpointed by the fact that under multiple pulse irradiation, the sample gets severely damaged even at the periphery of the beam, where the intensity is very low: note the large crater at the bottom of Supplementary Fig. 5, where the shutter was accidentally opened for several seconds, allowing about 30-50 pulses through at a moderate average pulse energy of about 2 μJ.

The primary mechanism for the damage observed is photo-mechanical spallation [3] of the 'low bandgap' GaP layer beneath the high bandgap HSQ layer (~9 eV bandgap SiO_2, requiring an order of magnitude more intensity to cause damage [4]), induced by the highly excited free-carriers in GaP. In such cases, it is our understanding that the topmost part of the GaP layer undergoes ultrafast melting [5] and rapid outward expansion [6], launching the HSQ layer with it [7]. This is what is observed in the region (ii). In region (i) with a higher threshold intensity, where the entire GaP layer undergoes ultrafast melting, ablation, and expansion, launches the entire structure, leaving the bare substrate. None of the cases exhibit any damage to the HSQ layer, pointing strongly towards ultrafast damage and ablation of the GaP layer as the main cause of the damage observed here under single pulse irradiation.

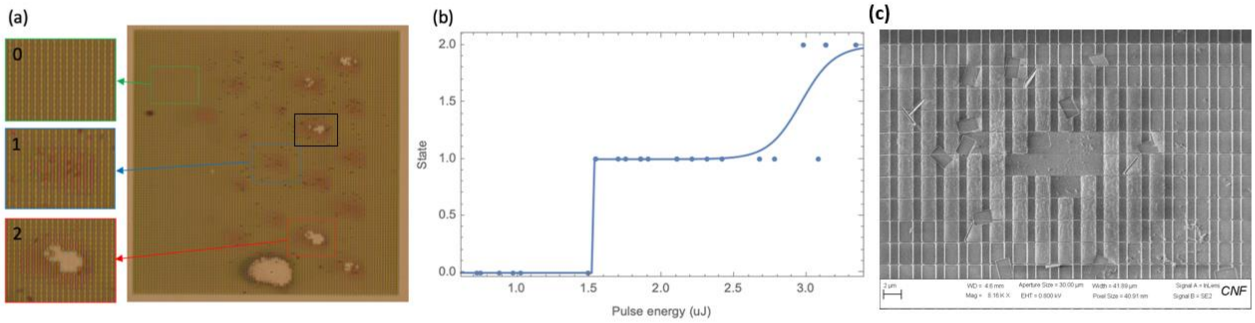

Supplementary Fig. 5 | Cases of single-pulse damage in GaP metasurfaces. a, An *a posteriori* optical image of sample #5 (resonant case), showing the different scenarios of pulse-metasurface interaction: from no damage at low fluences (scenario '0') to HSQ mask damage (scenario '1') to structural damage (scenario '2'). In '1,' the HSQ mask is detached from the surface of the sample, leaving the GaP resonators intact; the detached HSQ mask patches can be seen scattered around the sample as black dots. Scenario '2' describes a partial removal of the resonators in the center of the beam, with the bare substrate visible underneath. **b,** Damage threshold measurements. The dependence of the scenario (final state) number on the pulse energy shows two transitions characterized by the onset of mask detachment (around 1.5 μJ) and partial resonator ablation (around 2.5 μJ). The solid line is a double-logistic fit of the experimental data given with blue dots. **c,** SEM image of a damaged spot (black rectangle in panel **a**). HSQ mask detachment is seen at the anterior of the beam spot and full GaP metasurface detachment is observed at the interior of the beam. Redeposited HSQ and GaP particles can be observed.

7. Conversion efficiency estimates

A source of cw radiation at $\lambda_{\text{cw}}^{(1)} = 532$ nm, with a measured power of $P_{\text{cw}}^{(1)} = 80$ µW right after the focusing lens, was attenuated by a stack of neutral density filters with the measured transmittance of 0.11 (OD1 filter), 0.01 (OD2 filter) and 7×10^{-5} (OD4 filter), with the combined attenuation of $T^{(1)} = 7.7 \times 10^{-8}$, yielding the overall power at the sample site of $P_{\text{cw}}^{(1)} T^{(1)} = 6.2$ pW. This beam then passed through the rest of the setup and was detected by the camera sensor. Under the exposure time of $t_{\text{cw}}^{(1)} = 1$ s, the camera yielded $C_{\text{cw}}^{(1)} = 1.4 \times 10^6$ counts at the zeroth diffraction order. Therefore, assuming the linearity of the signal over the exposure time, the sensitivity of the detection system can be estimated at $S^{(1)} = C_{\text{cw}}^{(1)} / P_{\text{cw}}^{(1)} T^{(1)} t_{\text{cw}}^{(1)} = 2.3 \times 10^{17}$ cts/J. The H7 ($\lambda_{\text{H7}} = 565$ nm) emitted from the sample area #5 yielded $C_{\text{H7}} = 5.2 \times 10^5$ counts per $t_{\text{H7}} = 1$ s exposure window, which includes only the zeroth diffraction orders; other orders have, both reflected and transmitted, not been taken into account in the efficiency calculations. The 7th harmonic power collected from the sample is therefore $P_{\text{H7}} = \frac{C_{\text{H7}}}{S^{(1)} t_{\text{H7}}} \approx 2.3 \times 10^{-12}$ W. The average MIR power used in the experiment was equal to $P_{\text{MIR}} = 1$ mW (2 µJ per pulse at a repetition rate of 500 Hz); the conversion efficiency of H7 is estimated calculated as $\eta_{\text{H7}} = \frac{P_{\text{H7}}}{P_{\text{MIR}}} \approx 2.3 \times 10^{-9}$. From the relative intensities of the harmonics of different orders given in Fig. 2b, taking into account the spectral response of the detector (princetoninstruments.com/products/pixis-family/pixis) and objective (https://www.edmundoptics.com/p/20x-mitutoyo-plan-apo-infinity-corrected-long-wd-objective/6625/), we can estimate the following values for the conversion efficiencies of other harmonics: $\eta_{\text{H4}} = 1.7 \times 10^{-10}$, $\eta_{\text{H5}} = 2.6 \times 10^{-8}$, $\eta_{\text{H6}} = 1.0 \times 10^{-11}$, $\eta_{\text{H9}} = 2.1 \times 10^{-12}$.

A similar procedure was carried out in the single-pulse case. Here, the calibrating laser was used at $\lambda_{\text{cw}}^{(2)} = 633$ nm, close to the wavelength of the fifth harmonic ($\lambda_{\text{H5}} = 790$ nm). The cw power measured in the focal plane of the focusing lens was $P_{\text{cw}}^{(2)} = 166$ μW before attenuation. The attenuating filters in use were OD1 and OD6 with the measured combined transmittance of $T^{(2)} = 6 \times 10^{-8}$ and $P_{\text{cw}}^{(2)} T^{(2)} = 10$ pW of cw power in the focal plane. A $t_{\text{cw}}^{(2)} = 100$ ms exposure yielded $C_{\text{cw}}^{(2)} = 2.5 \times 10^5$ counts at the camera. The detection system sensitivity can be estimated at $S^{(2)} = C_{\text{cw}}^{(2)} / P_{\text{cw}}^{(2)} T^{(2)} t_{\text{cw}}^{(2)} = 2.5 \times 10^{17}$ cts/J. A single-shot exposure of the sample to a pulse with an energy of $E_{\text{MIR}} = 1$ μJ yielded $C_{\text{H5}} = 2.6 \times 10^5$ counts of H5 signal, totaling $E_{\text{H5}} = \frac{C_{\text{H5}}}{S^{(2)} t_{\text{H5}}} = 10^{-12}$ J of detected H5 energy. The conversion efficiency can therefore be estimated as $\eta_{5\omega} = \frac{E_{\text{H5}}}{E_{\text{MIR}}} = 10^{-12}\ \text{J} / 10^{-6}\ \text{J} = 10^{-6}$; adjusting for the spectral sensitivity of the camera and the objective at 633 nm and 790 nm results in $\eta_{5\omega}^{\text{adj}} = 1.4 \times 10^{-6}$ which almost two orders of magnitude larger than that of the multi-pulse case. Supplementary Table 2 provides a comparison between the conversion efficiencies for the 5th and the 7th harmonics in various solid-state HHG systems.

Material	Harmonic order	Conversion efficiency	Efficiency per 1 μm thickness
GaP metasurface [this work]	5 (SP)	1.4×10^{-6}	3.5×10^{-6}
	7 (MP)	2×10^{-9}	5×10^{-9}
ZnO [8]	5	3×10^{-5}	10^{-7}
	7	6×10^{-6}	2×10^{-8}
Periodically poled $LiNbO_3$ [9]	5	10^{-2}	4×10^{-7}
	7	10^{-2}	4×10^{-7}
Si metasurface [10]	5	5×10^{-9}	2.2×10^{-8}
ENZ material [11]	5	10^{-8}	1.3×10^{-7}
	7	10^{-10}	1.3×10^{-9}

Supplementary Table 2 | 5th and 7th harmonic conversion efficiencies in various reported solid-state HHG systems. GaP metasurfaces (this work) is represented by single-pulse (SP) and multi-pulse (MP) measurements.

8. Tensor analysis of harmonics' properties

Owing to the stark difference in the nonlinear susceptibility tensor structures for the even- and odd-order harmonics, their emission efficiencies and polarization states can be strongly dependent on driver polarization and crystal orientation. Figure 2d of the main text shows the intensity of H7 and H6 as a function of analyzer angle for one of the diffracted orders, with the analyzer placed after the collection objective. While H7 is polarized along the pump radiation (90°/270° direction), H6 is elliptical, with the main semi-axis directed at around 45° with respect to the pump polarization. Also, Fig. 2b shows disproportionality between the even and odd harmonics' intensities. The difference in the polarization response and conversion efficiencies between the even- and odd-order harmonics can be qualitatively explained in terms of the nonlinear tensor symmetries. For the odd-order processes:

$$\chi^{(2k+1)}_{i_1 i_2 \dots i_{2k+1}} \neq 0;\ i_1 = i_2 = \cdots = i_{2k+1},$$

meaning one can reasonably expect the main contribution from terms $I^{(2k+1)} \propto \chi^{(2k+1)}_{i_1 i_2 \dots i_{2k+1}}$. However, due to the zincblende crystal structure of GaP, in the bulk for the even-order processes,

$$\chi^{(2k)}_{i_1 i_2 \dots i_{2k}} = 0;\ i_1 = i_2 = \cdots = i_{2k},$$

meaning the main contributions to even-order harmonics will come from multiple off-diagonal components, opening potential to various polarization states of the output harmonics. We have analyzed the symmetry-enabled components of zincblende crystal nonlinear susceptibility tensors up to the 6th order; see Supplementary Code 1. In the configuration where the input field is in the form of $E = \frac{E_0}{\sqrt{2}}(1,1,0)$ in the frame of the crystal structure, the nonlinear polarization for odd-order processes is in the form of $P_{\text{odd}} = \frac{P_{\text{odd},0}}{\sqrt{2}}(1,1,0)$, where E_0 and $P_{\text{odd},0}$ are constants. In contrast, for even-order processes, the polarization is in the form of $P_{\text{even}} =$

$P_{\text{even},0}(0,0,1)$, where $P_{\text{even},0}$ is a constant. This means that if the GaP (100) plane were to lie in the plane of the metasurface, the nonlinear polarization at even harmonics would have been oriented out of metasurfaces' plane and weakly coupled to the zeroth diffraction order. Because of the 15° tilt of the normal to the metasurfaces' plane toward the [111] direction of GaP, some of this polarization outcouples to the zeroth order and can be detected. Nevertheless, the efficiency of this process is not optimized, and this fact serves to explain the low relative efficiency of the even harmonics in our experiment. Both the even- and odd-harmonic efficiency can be enhanced by judiciously choosing the crystal structure orientation. In Supplementary Fig. 6, the absolute values of the fifth and sixth order nonlinear polarizations, as well as their projections on the GaP crystal structure frame (see panel **a** for designations), are plotted as a function of the tilt angle θ ($\theta = 15°$ in the experiment). Here, for the sake of simplicity, we assumed that for all nonzero tensor components, $\chi^{(5)}_{ijklmn} = \chi^{(5)}_0$ and $\chi^{(6)}_{ijklmno} = \chi^{(6)}_0$. Panels **b**, **c** show the nonlinear polarization components generated in the metasurfaces along the x, y and z for H6 and H5, respectively. Changing θ from $15°$ to about $90°$ can boost the even harmonic output by almost two orders of magnitude, as well as the odd harmonic output by a factor of 17. The polarization state differences of the even and odd harmonics can be qualitatively understood as well. For the odd-order processes, at $\theta \approx 0°$ the major contributors to the emitted harmonics' polarization state are P_x and P_y components, where $P_x \approx P_y$, generating harmonic beams with approximately the same polarization state as the pump beam. In the even harmonic case, at $\theta \approx 0°$, $P_x \approx P_y \approx 0$, and P_z becomes the primary polarization. As θ increases, the contributions from all three directions may become similar in amplitude, generating polarization states that are elliptical and may not necessarily be aligned with the pump polarization, as seen in Fig. 2d of the main text. The interplay between the relative orientations of the crystal lattice and the

metasurface lattice is a promising topic of the future studies.

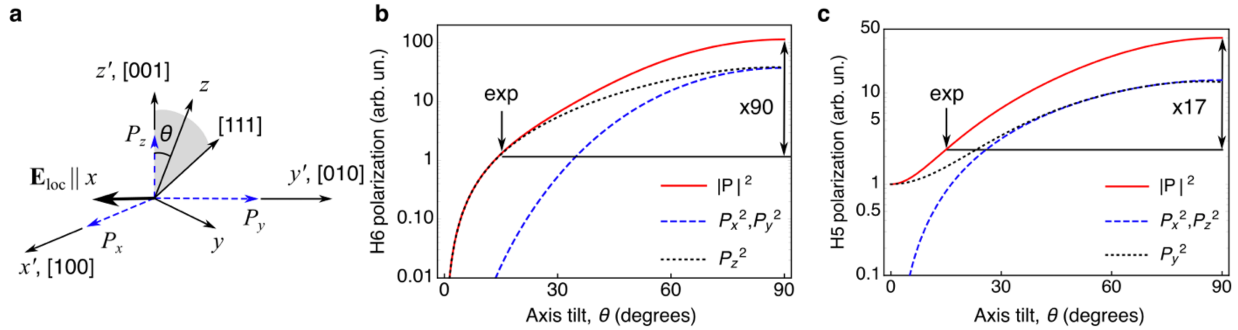

Supplementary Fig. 6 | Optimizing the HHG output by a judicious choice of the crystal lattice orientation in GaP metasurfaces. a, A model of local fields within the metasurface $\boldsymbol{E}_{loc}$ in the GaP crystal structure frame (x', y', z'). The direction of the average local fields within the metasurface coincides with the x direction in Fig. 1 of the main text. **b**, **c**, Polarizations of H6 and H5, respectively, as functions of the tilt angle θ between the normal to the metasurfaces' plane (z) and [111] direction of the GaP lattice. Solid red lines correspond to the total polarizations; the dashed blue lines correspond to polarizations along x' and y' (in **b**) and along x' and z' (in **c**); the dotted black lines correspond to polarizations along z' (in **b**) and y' (in **c**). The experiments were conducted for $\theta = 15°$. An overall enhancement of the nonlinear polarization by a factor of 90 for the 6th harmonic and by a factor of 17 for the 5th harmonic is observed if $\theta = 90°$.

Supplementary references:

[1] Perelomov, A. M., Popov, V. S., Terent'ev, M. V. Ionization of atoms in an alternating electric field. *Sov. Phys. JETP* **23**, 924 (1966).

[2] Keldysh, L. V. Ionization in the field of a strong electromagnetic wave. *Sov. Phys. JETP* **20**, 1307 (1965).

[3] Gupta, V., Argon, A. S., Cornie, J. A. & Parks, D. M. Measurement of interface strength by laser-pulse-induced spallation. *Mater. Sci. Eng. A* **126**, 105–117 (1990).

[4] N. Talisa, A. Alshafey, M. Tripepi, J. Krebs, A. Davenport, E. Randel, C. S. Menoni, and E. A. Chowdhury Comparison of damage and ablation dynamics of thin film interference coatings initiated by few cycle pulses vs longer femtosecond pulses. *Opt. Lett.* **45**, 2672 (2020).

[5] D. R. Austin, K. R. P. Kafka, Y. H. Lai, Z. Wang, C. I. Blaga, and E. A. Chowdhury Femtosecond laser damage of germanium from near- to mid-IR wavelengths. *Opt. Lett.* **43**, 3702–3705 (2018).

[6] C. Wu and L. V. Zhigilei, Microscopic mechanisms of laser spallation and ablation of metal targets from large-scale molecular dynamics simulations. *Appl. Phys. A* **114**, 11–32 (2014).

[7] N. Talisa and E. A. Chowdhury, Few cycle pulse laser ablation study of single layer TiO2 thin films using time resolved surface microscopy. *Opt. Express* **26**, 30371–30382 (2018).

[8] Gholam-Mirzaei, S. *et al.* High harmonic generation in ZnO with ahigh-power mid-IR OPA. *Appl. Phys. Lett.* **110**, 061101 (2017).

[9] Hickstein, D. D. *et al.* High-harmonic generation in periodically poled waveguides. *Optica* **4**, 1538 (2017).

[10] Liu, H. *et al*. Enhanced high-harmonic generation from an all-dielectric metasurface. *Nat. Phys*. **14**, 1006–1010 (2018).

[11] Yang, Y. *et al*. High-harmonic generation from an epsilon-near-zero material. *Nat. Phys*. **15**, 1022–1026 (2019).
