# Supplementary material for: Generation of even and odd high harmonics in resonant metasurfaces using single and multiple ultra-intense laser pulses
Source: Nat Commun. 2021 Jul 7;12:4185. doi: 10.1038/s41467-021-24450-9 (PMC8263774; doi:10.1038/s41467-021-24450-9)
Supplement: Supplementary file 2 — Description of Additional Supplementary Files [file 41467_2021_24450_MOESM2_ESM.pdf]

Description of Additional Supplementary Files

File Name: Supplementary Code 1
Description: This is a Wolfram Mathematica code to establish the relationships between various nonlinear susceptibility tensor components in zincblende crystals. Changing the generation matrices, as borrowed from the Ervin Hartman's pamphlet 'An Introduction to Crystal Physics,' University College Cardiff Press (2001), one can get similar relationships for other crystal classes. Nonlinear susceptibility orders 2, 3, 4, 5, and 6 are covered.
